# Impact of inter-ethnic anatomical findings on current surgical outcome, established biomarkers, and future artificial intelligence algorithms

**DOI:** 10.1007/s00417-022-05951-9

**Published:** 2023-01-06

**Authors:** Carsten H. Meyer, Sandeep Saxena

**Affiliations:** 1 Macula Center Grischun, Bahnhofstrasse 4, CH-7000 Chur, Switzerland; 2 Department of Ophthalmology, Philipps Univeristy, Marburg, Germany; 3 Department of Ophthalmology, King George’s Medical University, Lucknow, India

Dear Editors,

Laviers et al. investigated in a multi-center study the impact of ethnicity on post-operative anatomical closure rate following macular hole surgery and determined that Black patients had an approximately twofold lower anatomic success rate than White patients [1]. Anatomic failure occurred in 38.5% of Black patients versus 12.6% of White patients, while this difference remained statistically significant despite adjustment for predefined demographic or socio-economic status as well as confounding anatomical variables.

Since the early beginning of optical coherence tomography (OCT), we learned that the neuroretinal anatomy of the foveola may vary within certain conditions, e.g., foveal hypoplasia in oculocutaneous albinism [2]. Previous studies also determined a variation in the foveal morphology among different ethnic cohorts: the eyes of Black patients presented on OCT a larger, deeper, and broader foveal pits associated with a thinner central foveal neuroretinal thickness compared to White and Asian counterparts [3]. Murphy et al. found that even the size in unilateral macular holes correlated with the foveal floor of the fellow eye [4].

In addition, more recent OCT angiography (OCTA) investigations [5] also demonstrated a greater variability in quantitative measures of the retinal microvasculature among racial and ethnic groups: Hispanic, Black, and Asian patients had larger FAZ areas, in comparison to White participants. These differences persisted, even after adjusting for gender, age, and status in comparison to White patients.

These important neuroretinal and vascular OCT findings suggest that race and ethnicity may be important factors influencing established surrogated biomarkers [6], when evaluating the foveal morphology of healthy and diseased retina. The impact of these inter-ethnic findings is yet to be explored; however, it is conceivable that normative OCT parameters, biological determinants, and established threshold biomarkers may vary among different race and ethnicity groups and needed to elucidate in greater details to establish precise algorithms of future artificial intelligence systems.
